# Beirut Port Blast: Use of Electronic Health Record System During a Mass Casualty Event

**DOI:** 10.5811/westjem.20942

**Published:** 2024-11-27

**Authors:** Eveline Hitti, Dima Hadid, Miriam Saliba, Zouhair Sadek, Rima Jabbour, Rula Antoun, Mazen El Sayed

**Affiliations:** * American University of Beirut, Department of Emergency Medicine, Beirut, Lebanon; † McMaster University, Health Policy PhD Program, Department of Health Research Methods, Evidence, and Impact, Hamilton, Canada

## Abstract

**Introduction:**

Emergency departments (ED) play a central role in defining the effectiveness and quality of the overall hospital’s mass casualty incident (MCI) response. The use of electronic health records (EHR) in hospital settings has been rapidly growing globally. There is, however, a paucity of literature on the use and performance of EHR during MCIs.

**Methods:**

In this study we aimed to describe EHR use, as well as the challenges and lessons learnt in response to the 2020 explosion in the Port of Beirut, Lebanon, during which the hospital received over 360 casualties.

**Results:**

Information technology support, reducing EHR system restrictions, cross-function training, focus on registration and patient identification, patient flow and tracking, mobility and bedside access, and alternate sites of care are all important areas to focus on during emergency/disaster response planning.

**Conclusion:**

Innovative solutions that help address logistical challenges for different aspects of the disaster response are needed.

Population Health Research Capsule What do we already know about this issue?

*Hospitals and EDs face major operational challenges during mass casualty incidents (MCI). Research on the use of electronic health records (EHR) during MCIs is limited.*

What was the research question?

*This study examines EHR use during the Beirut port blast (August 2020) and highlights challenges and lessons learnt from this MCI.*

What was the major finding of the study?

*Opportunities identified for improved EHR use during MCIs included staff training and scaling up routine workflows to enhance efficiency of response.*

How does this improve population health?

*Hospitals adopting proposed EHR changes can better respond to MCIs, enhancing efficiency and leading to improved patient outcomes.*

## INTRODUCTION

Emergency departments (ED) play a central role in responding to mass casualty incidents (MCI) and define the effectiveness and quality of the overall hospital response.^1^ ^,^ ^2^ Hospitals must be prepared to respond to a large influx of patients and coordinate resources accordingly. This becomes particularly challenging in systems that lack a national or regional emergency medical services (EMS) plan and a disaster response framework.

Preparedness for MCIs is multifaceted and entails a multidisciplinary and integrated approach to develop a response plan that allows for prompt activation of a disaster code and notification of staff, expansion of triage and clinical areas, quick registration, rapid disposition, patient tracking, resources coordination, crowd control, efficient internal and external communication, and effective leadership and governance. Numerous previous studies have examined MCI responses; however, limited data exists on the performance of electronic health records (EHR) during MCIs.^3^ ^–^ ^5^

The adoption of EHRs by healthcare organizations has seen rapid growth globally, fueled by data supporting their positive impact on safety, quality and efficiency. The EHR offers valuable advantages in terms of strengthening patient tracking capabilities, improving clinician efficiency through quick access to medical records, and improving clinical decision-making through best practice advisories, as well as empowering healthcare systems with analytical and reporting capabilities.^6^ ^–^ ^13^ At the same time, some studies have raised concerns around declining throughput, lengthy documentation requirements, increasing complexity of work, and challenges with user-friendliness, as well as the level of available technical support for clinicians.^14^ ^–^ ^21^ This is particularly relevant to ED settings where time constraints and complex processes pose additional challenges.

Use of EHRs in MCIs requires the development of specialized disaster modules that involve pre-registered records that can be quickly accessed and activated in a disaster. Given the time constraints in MCIs, where routine processes might not keep up with the flow of patients, specialized workflows around documentation, financial clearance, computerized physician order entry (CPOE), medication management, patient tracking, and admission and discharge are required.^12^ ^,^ ^22^ To our knowledge there are no peer-reviewed publications documenting the use and performance of EHRs during live MCIs.

On August 4, 2020, during the COVID-19 pandemic, Lebanon witnessed its largest non-conflict-related MCI, the Beirut port blast, which left behind ≈200 dead and 6,500 injured.^23^ In this study we aimed to describe EHR use during the MCI response, as well as highlight the challenges and lessons learnt in response to the Beirut port blast, one of the largest non-nuclear explosions in history.

## METHODS

### Design

This is a case report with a review of the literature. We searched both Pubmed and MEDLINE using the following keywords: electronic medical record; disaster planning; mass casualty incident; hospital emergency preparedness; and medical informatics. This resulted in the identification of three manuscripts. All three involved limited use of information technology (IT) in a MCI, and only one involved a live activation.^24^ ^–^ ^26^ The search did not yield any manuscripts on MCI response with a fully integrated EHR system.

### Facility

The American University of Beirut Medical Center (AUBMC) is the largest academic, tertiary-care center in Beirut with over 57,000 ED visits annually. In the past 17 years, AUBMC-ED has been at the forefront of responding to over 15 MCIs. As a result, AUBMC regularly assessed and modified both its ED and hospital emergency preparedness plan (EPP). The AUBMC established its own EPP in July 2000. Yearly drills and modifications to the existing plan were conducted to ensure better communication, coordination, and availability of resources during MCIs. In 2019, AUBMC became an HIMSS Stage 6 Emergency Medical Record/EHR institution with the implementation of the Epic EHR (Epic Systems Corporation, Verona, WI).

### The Event

On August 4, 2020, at 6:07 pm, an estimated 2,750 tons of stored ammonium nitrate exploded at the Port of Beirut less than 2.5 miles from AUBMC.^27^ The Beirut port blast was reported to be one of the largest non-nuclear explosions in history leaving behind more than 6,500 people injured, approximately 300,000 displaced, and 204 fatalities.^27^ Casualties immediately flooded nearby hospitals, which had already been partly destroyed by the blast.

The AUBMC ED started receiving casualties from within the institution and from its neighborhood less than three minutes after the explosion due to its proximity to the blast site. The highest EPP notification level “Code D-Full Activation level” was immediately activated at AUBMC, where all hospital staff were notified to respond. During the three-hour interval following the explosion, over 360 victims were treated in the ED, of whom 87 required admission (52 regular bed admissions, 19 critical care cases, and 16 immediately operated on) and 12 reported dead upon arrival.

### Description of the Emergency Preparedness Plan

The disaster plan at AUBMC consists of two levels of MCI response (Code D): partial and full activation. Activation usually follows an alert notification “Code D Alert.” “Code D – Partial activation” requires all essential staff to report to their corresponding departments including the ED. “Code D – Full Activation” requires all active staff to report to their corresponding departments. Activation of the disaster plan and its corresponding response level is traditionally based on the geographical location of the incident and on the number of casualties expected to present to the ED.

The hospital director or administrator on call is in charge of announcing the level of hospital response. Code D Alert is usually announced by the ED director, chair, or delegate for MCIs located in a predefined geographical area surrounding AUBMC. Activation levels are communicated through short text message (SMS), paging and WhatsApp messages to pre-prepared disaster lists of hospital staff. This ensures redundancy of communication since cellular networks usually experience delays in SMS delivery during MCIs due to infrastructure damage and call overload on landlines and mobile networks.

Upon activation of the EPP, initial steps of the response consist of quickly transforming the ED physical space to designated color coded “surge areas,” with deployment of pre-prepared supplies and medication carts to different sections. A mobile triage area is set up and existing ED patients are discharged or sent to inpatient units to improve surge capacity and prepare for the first wave of casualties. The MCI patients are color tagged upon arrival to the ED and directed from triage to designated areas based on a color-coded triage system. Patient triage shifts from Emergency Severity Index scoring to the modified care-flight system. A detailed description of the plan has been previously published.^1^

### Emergency Preparedness Plan Post Implementation of an Electronic Health Record

During the implementation of Epic in 2018, scoping and adoption phases focused on including a streamlined disaster module because of the high frequency of MCIs in Lebanon. The foundational Epic disaster module that is part of the standard implementation package was modified for alignment with existing workflows. Final workflows that were integrated into the disaster module are summarized in Table 1. With the implementation of the EHR, the response plan shifted from pre-prepared manual charts to a full electronic system using pre-printed bracelets containing pre-assigned mass casualty record (MCR) numbers and requiring barcode scanning for activation. The MCR numbers are unique identifiers that allow identification and tracking of casualties during the disaster response phase.

**Table 1.** Changes done to emergency preparedness planning after introduction of an electronic health record.

Elements	Pre-EHR	Post-EHR
Communication display of a disaster status on the dashboard	Not available	ED nurse manager and/or ED charge nurse and/or ED clerks activate the ED department status to “Disaster” on the ED dashboard.
Registration of casualties	Registration is performed manually.Pre-labeled emergency wristbands are used at the triage area.	Pre-printed identification bracelets available with the PA team, are used during partial and/or full activation of the disaster.Pre-printed bracelets contain preassigned MCR numbers and patient hospital number.PAOs provide each casualty with a bracelet, scan it, and activate the chart in EPIC.Once the casualty is registered, the record appears in the Disaster section, the triage RN then completes the triage using the Disaster Navigator and assigns the casualty to an ED section.
Identification of casualties	MCR numbers are used as the only tracking reference for all concerned departments. The patient’s name, if identified, is shown in another field visible to ED and PA staff.	MCR numbers are the only tracking reference for all concerned departments. Patient’s name when identified is added to Aliases and not to the primary name field.
Tracking of casualties	Casualty tracking checklists are used. These included:• MCR number • Chief complaint • Entry/exit to/from the color-coded areas • Comments • Patient disposition	The “disaster view” is used to track casualties’ movements during disaster, using EPIC disaster navigator.Disaster reports are generated at any point in time during a disaster to track casualties.
Discharge process	Pre-labeled discharge form is used to document diagnosis and follow-up instructions.	Full demographic data are collected by PAOs before discharge. PAOs collect the information included in the ED disaster discharge checklist (MCR number, patient triple name and phone number), to ensure that the patient is registered in the EHR and to provide the patient with the discharge instructions.
Documentation	A pre-labeled kit system is used in the triage area. These kits contain pre-labeled emergency paper charts and preassigned MCR numbers.	During a disaster, the EHR is used by the medical and nursing teams.Nurses and physicians use “disaster navigator” with minimal documentation.
Ordering	Pre-labeled emergency studies’ requests are used in the color-coded areas.	All orders required during disaster (laboratory, medications, radiology, procedures, admissions, etc), are ordered through the disaster orders navigator.An admission order is also placed by the medical team for casualties requiring admission.
Radiology process	Pre-labeled emergency studies’ requests are used.A radiologist reports the major findings of the radiograph, or CT requests handwritten.	All radiology studies are ordered through the disaster orders navigator, and results are reported on the AGFA/EHR system.
Laboratory process	Pre-labeled emergency paper orders are used.Lab results are communicated verbally to the ED team by phone with read back or handwritten on the lab requests and sent to ED by pneumatic tube.	Rainbow draws are ordered by the RNs on all patients admitted to red and yellow areas, unless the MD already placed orders for these patients.ED EMTs /RN print labels, collect the samples using rainbow draw process, and send them to receiving area using pneumatic tube.MDs, in parallel, order the required tests using the EHR.Laboratory then proceeds with testing, and the results appear electronically on the patient’s chart.
Charging and financial clearance	Charging is performed manually. Casualties are assigned a specific guarantor (070).All charging documents are given by the ED team, at the end of the disaster to the ED cashier.The ED cashier logs them on the billing system (AS400) after the patient’s discharge.	Casualties are automatically assigned a specific guarantor (070), thereby allowing automatic clearance of ED registration.Charging is performed electronically using the EHR, during or after the patient’s stay. All charges are cleared by the cashier on the billing system (AS400).
Recovery process	Upon disaster termination, all casualties are manually entered on the dashboard, to complete their registration on AS400, placing the necessary charges, and admission orders, if needed, and discharging them.	Upon disaster termination, a recovery process is initiated to update information about each casualty in the EHR (registration status, location, and disposition).
Medication dispensing during disaster	Emergency medication carts are used (Pyxis did not exist).	Emergency medication carts are used. Medications are also dispensed using the ED Pyxis machine. Pyxis is replenished from the central pharmacy.
Equipment	Paper system (workstation wheel [WOWs] did not exist).	ED and PAO staff use WOWs in the triage area, inside the ED, and at discharge

*ED*, emergency department; *EHR*, electronic health record; *PA*, patient access; *PAO*, patient access officer; *Pyxis*, automated medication dispensing system; *RN*, registered nurse; *MCR*, mass casualty record; *CT*, computed tomography; *MD*, medical doctor; *EMT*, emergency department technician; *WOW*, workstation on wheels.

Smart groups of preferred orders (laboratory, medication, radiology, admissions, procedures, etc) were built into the disaster module based on previous data from MCIs. These would be requested electronically in Epic when the disaster module is activated. Results are viewed electronically when available, which would allow physicians to access real-time patient data.

Tracking and identification of casualties also shifted from a paper-based system to an electronic system whereby staff and caregivers can track casualties at any point in time on “disaster view” using the EHR disaster navigator. Moreover, charging and financial clearance in the disaster module are performed automatically instead of manually. Documentation within the disaster module included a simplified trauma template. The EHR implementation plan also included allocation of additional workstations on wheels that can be deployed into the surge areas for use by front-liners. Additional areas were designated for workstation deployment and included triage and the low-acuity “green area,” as well as the discharge area.

### Debriefing

After the MCI, several debriefing sessions were held to review strengths and areas for improvement in our response protocols. Psychological debriefing sessions were conducted within 72 hours of the event with all team members who actively participated in the incident including faculty, residents, and nursing teams. These initial debriefings were led by the chair of the department. They were carried out immediately after the incident for staff who were involved directly in the MCI response and focused on emotional support, leaning on principles outlined in the MCI CORD (Council of Residency Directors in Emergency Medicine) Survival Package for psychological debriefs.^28^ The psychological debriefing sessions were subsequently followed by technical debriefs conducted around three weeks post event by a multidisciplinary team including clinical and non-clinical staff from the ED (chair of ED, ED medical director, ED nurse manager, ED quality officer), surgical department (chair of surgery, director of trauma), medical informatics team (director of medical center applications, and chief medical information officer), risk management (director of quality safety and risk management, and safety officer), nursing team (director of nursing), and patient registration team (director of patient registration).

The technical debriefs were led by the director of quality safety and risk management and followed the after-action review framework, including a review of the incident, an analysis of both the successes and challenges encountered, as well as an identification of root causes of any shortcomings.^29^ Minutes of the debrief sessions were recorded by the quality team and sent to participants for review, comments, and approval prior to finalization. These minutes were then analyzed by two of the authors following the six-stage process for thematic analysis.^30^ This included data familiarization, initial code generation, reviewing themes, defining and naming themes, and completion of write-up. Through this process, we identified several key focus areas. Key findings and discussion points raised in our debriefing sessions were used to modify the plan for future responses. Since this event was the first to test the EHR component of the EPP plan, a major part of the debriefings focused on EHR-specific issues including workflows and the performance of the EHR’a different features.

## RESULTS

### Emergency Preparedness Plan Response

We identified several effective elements of EPP response. The actual treatment areas rapidly expanded to designated “surge areas” within and outside the ED. Patient triage rapidly shifted to the modified care-flight system. Non-critical casualties were directed to pre-designated low-acuity or green surge areas with adequate medical teams and supply carts. The influx of patients in terms of rate and number presenting to the ED within the first hour was, however, much higher than expected due the proximity of AUBMC to the explosion site. Casualties were treated in hallways and at the ED entrance on stretchers, chairs, and floors. Treatment of patients proceeded in a quick manner as in previous MCIs with most hospital staff responding immediately upon activation.

### Challenges

Numerous issues were, however, faced with the EHR during the response.

### Workflow Related

An initial delay occurred in activating the disaster status on Epic since it needed to be activated manually by specifically trained members who were not present physically in the ED at the time of the explosion. This prevented the appearance of the disaster navigator on the dashboard and delayed information relay to different stakeholders in the ED.

Patient registration was another main challenge. The EPP planned for 200 pre-prepared disaster e-records/wristbands. The number of casualties exceeded this within the first hour. Calling in additional registration staff caused delay in registration and inability to capture demographic information for patients who were discharged early, in addition to delays in ordering tests and placing orders on other patients. Creating additional records was logistically challenging since it required both registration staff and IT support. Loading all the records within a short time interval also slowed the system in terms of response. Back-up manual charts were, therefore, used until additional wristbands were printed and corresponding electronic records were created. While MCRs were used as unique identifiers, this prevented easy identification of casualties since registration staff were not collecting patients’ names or demographic data when handing out MCR bracelets.

Issues with patient flow and with financial workflows were also identified. Beyond operational difficulty in managing the high number of admissions to hospital, the staff experienced additional challenges related to complex workflows that required following routine admission process for every patient. This proved to be difficult especially for physicians responding in the ED since they were focused on patient care rather than completing steps in admission workflows to allow for patients to transition electronically to inpatient units. Eighty-seven patients required admission, and most of them were physically sent to inpatient units prior to completing the extensive admission electronic process. Financial constraints were also identified for patients during their transition to inpatient status despite previous modification of financial workflows for the ED in the EPP. Charges were not automatically waived in the operating room or inpatient for patients, similar to what happened at the level of the ED, which required cashiers to manually clear charges during the event.

Medications and supplies were dispatched immediately to different treatment areas in the ED as planned in the EPP. Carts were managed by pharmacists, nurses and store staff who used paper logs while helping dispense different items. Routine medication from Pyxis (the automated medication dispensing system) and supplies workflows were, therefore, bypassed during the event.

### Real-time Operations

Patient tracking was also another challenge faced during this MCI response. Several clinical and administrative staff were tracking the number of casualties arriving to the ED; however, moving patients on the dashboard between different sections was delayed, which resulted in difficulties in tracking casualties and in identifying their exact physical location.

### Training Related

Clinical documentation was suboptimal during the event. Despite introducing a streamlined one-page “express lane” disaster-documentation module, limited clinical documentation occurred for casualties treated in the ED. Key challenges were related to available workstations, as nearly all areas in the ED were transformed into clinical areas given the high number of casualties. Despite previous training, physicians from other units were not familiar enough with the documentation process on ED patients during EPP. Many patients were discharged physically from the ED with minimal or no documentation.

### Recovery Process

Once CODE-D was deactivated, recovery operations resumed. These consisted of cleanup of ED clinical areas, reconciliation of different casualty lists and fatality management/identification, in addition to resupplying essential equipment and restocking ED medications. The Epic dashboard recovery process was not previously planned; therefore, this required setting up a clinical/IT group to help clean up the ED dashboard and to resolve tickets related to patient flow, CPOE, and disposition-related issues (Table 2).

**Table 2.** Summary of challenges and lessons learned during the response.

Process	Events and challenges	Lessons learned
Workflow related	Disaster plan activation on the EHR was delayed as it needed to be manually activated by specific trained members: • Prevented the appearance of disaster navigator• Delayed information relay to different stakeholders in EDDelay in Patient Registration: • The number of casualties arriving to the ED exceeded the number of pre-printed disaster wristbands (200). This required additional printing of wristbands, which was logistically non-workable. • Calling in the registration staff delayed patients’ registration caused delay in registration and inability to capture demographic information for patients who were discharged early in addition to delays in ordering tests and placing orders on other patients. • Creating additional records delayed as it required both registration staff and IT support. • Use of MCRs prevented easy identification of casualties since registration staff were not collecting patients’ names or demographic data when handing out MCR bracelets.• Printing of MCR wristbands delayed due to limited number of patient access users.Delay in patient admission on the system: • Patients were sent to inpatient units prior to completing the extensive admission electronic process.Financial constraints identified for patients during their transition to inpatient status: • Admission financial clearances were not automatically overridden in the operating room or inpatient for patients, similar to what happens at the level of the ED, which required patient access to manually clear the admission requests during the event.	• Ready bracelets with unique identifiers allow for quick activation of records once patients arrive to ED. • Improved workflows on handheld devices to help streamline patient registration/ identification/ care during MCI events. • Alternative methods of health records activation should be implemented, which include using dormant records with unique identifiers activated by scanning wristbands, as well as pre-printed back up manual charts. • Activation of these records can be done by registration and by nursing staff at triage and at bedside if needed until the registration staff scale up in terms of response. • Training on activating disaster status on the EHR should be done for different ED staff and not limited to nurse managers during MCIs. • All staff should be cross trained on performing different tasks related to EHR interface during MCIs. • Having members of the IT staff on the ground relaying the technical issues back to the complete IT team is more effective. • Access/roles limitations present in routine workflows should be addressed and bypassed if needed during an MCI. • Throughput restrictions related to financial clearance, patient admission/discharge/flow through phases of care should be reviewed and restrictions lifted to allow seamless patient transfer across the hospital.
Real-time operations	Inefficient patients tracking during and post disaster:• Delay in patient registration led to inconsistency in the number of patients registered on system with those registered on paper. • Several clinical and administrative staff were tracking the number of casualties arriving to the ED. This caused delay in moving patients on dashboard between different sections which resulted in difficulties in tracking casualties and in identifying their exact physical location.	• Triage in non-clinical areas, bedside registration by registration staff and nurses, collecting and recording information, CPOE and access to results are all important functions that can be done at bedside. • Maximize the use of handheld devices to help streamline patient care/ flow during MCI events.
Training related	• Despite previous training, physicians from other units were not very familiar with the documentation process on ED patients during EPP. • Many patients were discharged physically from the ED with minimal or no documentation.	• Recording the MCR numbers and demographics from patients prior to them physically leaving the ED is important to reconcile lists.• Unified electronic reports that show casualty lists with clear identifiers and demographic data should also be available to administrative and clinical staff in charge during the MCI response.
Recovery process	• Recovery process was not previously thoroughly planned. • It required setting up a clinical/IT group to help clean up the ED dashboard and to resolve tickets related to patient flow, CPOE and disposition related issues.	• Assess system responsiveness, address tickets related to workflows, onsite help with registration/CPOE/reconciliation issues.• Establish a trained multidisciplinary team to address tickets/resolve pending issues.

*ED*, emergency department; *EHR*, electronic health record; *EPP*, emergency preparedness plan; *IT*, information technology; *MCR*, mass casualty record; *MCI*, mass casualty incident; *CPOE*, computerized physician order entry.

## DISCUSSION

This review describes EHR challenges and lessons learned at an academic, tertiary-care center in Lebanon during the response to the Beirut port blast, which was one of the largest non-nuclear modern explosions in history, and reviews the literature on EHR use in a MCI. The main challenges in this case experience were related to complex workflows, training on EHR workflows, and the recovery process. Translatable lessons include improving workflows to streamline patient registration, identification, and care during MCIs. Cross-training of staff on patient-registration disaster modules is also important to capture the large influx of patients. In addition, having members of the IT staff on the ground addressing acute issues during an MCI is an important part of EPP preparedness using EHRs. While a few studies have reported on the use of technology in MCI, these have been limited to specific aspects of IT, such as radiology order entry and tracking systems. No studies to our knowledge have reported on the use and performance of a comprehensive EHR during an MCI.

Non-EHR essential concepts during MCIs focus on external and internal communication. External communication with EMS agencies, command centers, and other neighboring hospitals is needed to coordinate response and provide status updates on capacity and on dynamic readiness of hospitals to accept additional casualties when near a disaster event. Most hospitals in Beirut were affected to varying degrees by the explosion, and some became non-functional because of the damage. Two large hospitals very close to the explosion site needed to coordinate with EMS transfer of their patients who were already inpatients to other hospitals outside the Beirut area. Several casualties walked directly from areas near the explosion sites to the closest hospitals and found them to be non-functional.

Internal communication was also key and used various methods to notify all essential staff. Notification of staff and dispatch of resources and personnel was effective. Alerts used redundant communication SMS, paging, and WhatsApp notifications of members of pre-prepared disaster lists. As in prior events, infrastructure damage delayed SMS delivery in Beirut; however, pager messages and WI-FI messaging systems alerts were promptly received. This allowed for immediate dispatch of staff to the ED as well as the opening of additional treatment areas with distribution of pre-prepared supply and medication carts to all sections. This is in line with previous literature showing that effective intraorganizational communication is critical for crisis planning.^31^

The EHR-specific workflow challenges such as patient registration, identification, and tracking during MCI remain challenging. Ready bracelets with unique identifiers allow for quick activation of records once patients arrive to the ED. They allow for ease of ordering/results management during MCIs. They can also help with patient tracking and patient flow between the ED and various departments. However, patient identification can be difficult if demographic information is not collected immediately. Communication with search-and-rescue teams, media, and relatives of casualties required immediate identification of patients and their location (inside the hospital and at other hospitals) during the event; this proved to be initially very difficult especially for unconscious patients since identifying patients was a step previously assigned to the inpatient/pre-discharge phase of care. Casualty reports did not initially identify patients, which resulted in communication challenges. Additionally, closing the loop during the discharge process by recording the MCR numbers and demographics from patients prior to them physically leaving the ED is important to reconcile lists. Unified electronic reports that show casualty lists with clear identifiers and demographic data should also be available to administrative and clinical staff in charge during the MCI response. These enhancements can help close gaps in EHR adoption in the context of MCIs and address previously reported difficulties in this setting.^32^

Another EHR-related challenge was mobility during MCIs; use of handheld devices can help improve mobility of staff during response. Fixed workstations work during routine operations; however, with the need to expand treatment areas, the influx of casualties and medical staff responding to event, bedside access to EHRs is key. Triage in non-clinical areas, bedside registration by registration staff and nurses, collecting and recording information, CPOE, and access to results are all important functions that can be done at bedside. With the increasing number of casualties, dedicated staff were assigned to accompany and care for patients during their ED stay and to handoff essential information to other treatment teams. Such models of care require maximizing the use of handheld devices to help streamline patient care/flow during MCI events. Disaster- related plan for equipment/handheld devices should also be part of an emergency/disaster response plan.

The EHR access workflows need to be tailored to the MCI response. Addressing access and role limitations that are present in routine workflows for different provider types is also important. Routine quick registration and full registration workflows are not effective during a high influx of patients. Limitations are related to immediately available registration staff and to information collection requirements. Planning should be done for alternative methods of health records activation, which include using dormant records with unique identifiers activated by scanning wristbands, as well as pre-printed, back-up manual charts. While the EHR- based response within our institution had integrated these features, registration staff were the only team members trained on this step and were not able to keep up.

Cross-training of nursing staff at triage and at bedside if needed until the registration staff scale up in terms of response is essential. Moreover, training on activating disaster status on Epic should be done for different ED staff and not limited to nurse managers during MCIs. This step is key to activating a disaster module with corresponding disaster documentation. Additionally, cross-training all staff on performing different tasks related to EHR interface during MCIs is crucial. This should be accompanied by addressing access/roles limitations present in routine workflows and bypassing them if needed during an MCI. Examples would be to allow physicians or nurses to activate a patient registration event. Similarly moving patients electronically from one area to the next can be done by different clinician types. This role fluidity can enhance previously suggested models to optimize the management of patient flow and medical resources during MCIs.^33^

Testing admission/flow/discharge workflows in planning for MCIs is also key. Throughput restrictions related to financial clearance and patient admission/discharge/flow through phases of care should be reviewed and restrictions lifted for allowing seamless patient transfer across the hospital. With the high number of complex events occurring during an MCI, routine EHR-related required steps in different units/departments become very cumbersome and may result in unnecessary complications. For example, routine sequential steps do not allow a patient to be admitted to the intensive care unit (ICU) and for clinicians to initiate CPOE of care bundles in the ICU before a physician places an admission order in the ED.

The IT-related activities become extremely complex during MCIs. Scaling up quickly in terms of IT helpdesk and support is key during an MCI. Assessing overall system status, creating new records, addressing tickets related to workflows, and providing onsite help with registration/CPOE/reconciliation issues are very important. Setting up additional new treatment areas also requires IT support. The recovery phase also entails having a trained multidisciplinary team to address tickets/resolve pending issues. Business continuity activities (BCA) plans that are usually put in place do not account for operations during MCIs. During the Beirut port blast, the IT infrastructure remained intact. However, in the event where IT infrastructure is affected, EHR systems might be impacted, and institutions should resort to alternative BCA measures. Literature on IT support in such critical situations is limited. Future research will shed more insights on this aspect of MCIs.

## LIMITATIONS

This study has several limitations. The results reflect the experience of a single center using a specific EHR, limiting generalizability of our findings. At the same time, this is the largest medical center in Lebanon with one of the largest catchment areas. In addition, the EHR adopted at this center is one of the most widely used across the United States with a growing presence globally.^34^ Recall bias of participants during the debriefs and transcribing biases are additional limitations that may have impacted the findings that were documented in the minutes. However, the practice of sending minutes to all meeting participants for comments and approvals prior to finalization mitigates the latter.

## CONCLUSION

We have outlined the challenges of using an electronic health record system in a large-scale mass casualty event. The main improvement opportunities were related to staff training, failure to address the recovery process in the initial plan, and complex EHR workflows that failed to effectively scale up to the rapid influx of patients. In particular, streamlining EHR workflows related to patient registration, patient identification/ tracking, and patient admission is critical to handling the scale of patient flow during large-scale MCIs, as is staff training on time-sensitive registration processes. Addressing these challenges a priori in settings that rely on EHR use and incorporating on-the-ground IT support as part of the response team is essential to an effective hospital MCI response.
